# Deciding the fate of disputed embryos: ethical issues in the case of Natallie Evans

**DOI:** 10.1186/1743-1050-4-2

**Published:** 2007-07-04

**Authors:** Anna Smajdor

**Affiliations:** 1 Medical Ethics Unit, Imperial College London, UK

## Abstract

**Background:**

A number of disputes have arisen in recent years over the status of non-transferred embryos cryopreserved during in vitro fertilisation. One such case is that of Natallie Evans who in April 2007 lost her final attempt to prevent the destruction of embryos created with the sperm of her former partner. Ms Evans had been rendered infertile by cancer treatment, and the embryos represented her only chance of having genetically related children.

**Discussion:**

Arguments over stored embryos often conflate different concepts of parenthood. The effects of 'forcing' genetic parenthood on a man are mistakenly presented as being analogous with forcing women to bear children. Likewise, there is a tendency to assume that genetic parenthood necessarily involves legal, financial and psychological implications. Men (or women) who object to becoming parents should be encouraged to specify which aspects of parenthood they regard as being harmful. While the financial or physical burdens of forced parenthood involve objective harms, the putative psychological harms of enforced genetic parenthood are subjective, and this distinction should be recognised. Popular beliefs about genetic parenthood perpetuate the kinds of subjective concerns expressed by Ms Evans' partner, but the concept of genetic parenthood itself may come under pressure in the face of future technological developments.

**Summary:**

Historical legal requirements obliging men to provide for their genetic offspring still pervade in the law. These perceptions are becoming outmoded in context of rapidly-moving reproductive technologies. To avoid disputes greater flexibility is required. The economic and legal components of parenthood should be negotiable in cases where disputes arise, and should not be assumed to flow inexorably from genetic paternity. To reduce the chances of disputes arising, consent protocols for cryopreservation of non-transferred embryos should be refined. Couples should address the possibility of divorce or the breakup of their relationships, and should be made aware that embryos can be destroyed at the behest of either party in these circumstances.

## Background

On 10^th ^April 2007, a British woman, Natallie Evans, lost the final stage of a four year legal battle for the right to implant embryos created with her eggs and the sperm of her former partner[1]. Ms Evans had been diagnosed with ovarian cancer, and had been told that her ovaries would have to be removed in the course of treatment.

Ms Evans' relationship with her partner Howard Johnston subsequently broke down. Mr Johnston withdrew his consent to the storage or use of the embryos created with his sperm and Ms Evans' eggs. Since the consent of both parties is required for fertility treatment or even for ongoing storage of embryos, it seemed that Ms Evans would have to forego her dream of parenthood. However, she was unwilling to submit to the loss of her embryos without a fight. She embarked on a protracted legal struggle which culminated in the European Court of Human Rights' rejection of her case[2].

The issues raised by the case were widely reported in the media. Many people, while sympathetic to Ms Evans' plight, felt that the court had come to the right conclusion[3]. Clear consent protocols for the creation, storage and use of embryos are set out in schedule 3 of the 1990 Human Fertilisation and Embryology Act (the HFE Act)[4]. Both parties were made aware of the right of veto when the embryos were created. But while in the eyes of the law the correct decision may have been reached, the case raises some interesting questions.

The implication of the court ruling was that people should not be forced to become parents, other things being equal. I suggest that this conclusion was based on a false analogy that conflates genetic with gestational and other aspects of parenthood. I also argue that legal and social expectations are based on outmoded assumptions which no longer make sense in the context of new scientific developments. Finally, I query the adequacy of standard consent forms, and suggest some ways in which patients and clinics could minimise the danger of future recurrences of this kind of impasse.

## Discussion

The reasons for Ms Evans' failure to persuade the courts of her right to use the embryos rested on the idea that it would be wrong to force parenthood on a man who was unwilling to accept or assume that role. But if this is really so, we need to be sure of what is understood by parenthood. I suggest that parenthood is best understood not as inhering solely in genetic ties. Rather, it is a bundle of concepts which may include some or all of the following[5]:

• sharing genetic links

• undergoing gestation and childbirth

• being part of a causal chain that culminates in the birth of a child

• having the intention to procreate

• acquiring legal and financial responsibilities

• nurturing and rearing

Some of these aspects of parenthood should not be forced on unwilling people. But does this justify the outcome of the Natallie Evans case? To answer this question, we must examine the connections between those components of parenthood which are objectively harmful, and establish whether they are necessarily connected with the birth of genetically-related offspring.

### Genetic and gestational parenthood

Should we regard people as having a right not to be a parent? And if so, does this imply that people should not have *genetic *parenthood forced upon them? Margaret Brazier has suggested that rights should be gender neutral[6]. If there is a right not to be a genetic parent it ought therefore to apply equally to both sexes.

In fact men's supposed rights not to become genetic parents are routinely overridden. Once a woman is pregnant it is widely accepted that her partner cannot force her to undergo abortion. The unwilling father is simply obliged to accept the woman's choice. The greater weight given to the woman's choice demonstrates the importance we place on deciding what is done to our own bodies. But this does not necessarily spring from a right not to be a parent. It is more plausible to understand it as a right to self determination. This would fulfil Brazier's criterion of being gender-neutral, since it can be applied equally to men as well as women. However, it is not associated with anything specific to parenthood.

These points may seem obvious, yet the conflation of genetic and gestational parenthood was at the root of one of the primary arguments used against Natallie Evans in her first court case. Mr Justice Wall stated:

"If a man has testicular cancer and his sperm, preserved prior to radical surgery which renders him permanently infertile, is used to create embryos with his partner; and if the couple have separated before the embryos are transferred into the woman, nobody would suggest that she could not withdraw her consent to treatment and refuse to have the embryos transferred into her. The statutory provisions, like Convention Rights, apply to men and women equally"[7].

Mr Justice Wall suggests that if Ms Evans had used the embryos against her ex-partner's wishes, this would be analogous with his forcibly implanting them in her body against her will. Yet there is a hugely significant difference between the two scenarios. The latter involves an enforced implantation procedure followed by a coerced nine month pregnancy and childbirth or a forced caesarean. In the absence of consent, this would be regarded as a criminal assault. The former case involves no contact with the body of Mr Johnston at all.

The analogy also fails to recognise that if a man in the situation described above wanted to procreate, he could retain the possibility of doing so without imposing gestational parenthood on his unwilling ex-partner. That is, he could find a surrogate. Whether the female ex partner in such a situation could justify a complaint is precisely the question at issue, but the example above fails to shed any light on this.

### Genetic and legal/financial parenthood

Arguably, a woman in the situation described by Mr Justice Wall would have fewer grounds for complaint than a man. She would have no legal or financial responsibility for the child. In this context, British law is oddly asymmetrical. A woman's genetic link with a child confers no automatic parental responsibility by law, but a man's almost invariably does.

This inequality is compounded by the fact that neither the genetic nor the gestational mother of a child in the UK is obliged to accept responsibility for its maintenance or upbringing. A woman who gives birth to a child can relinquish any financial or legal responsibilities by choosing to place it for adoption. But the genetic father is not permitted to renounce his responsibility for the child in this way. In this respect women's advantages over men extend far beyond what can be justified solely by virtue of a physical connection with the offspring[8].

The development of reproductive technologies has enabled us to separate many of the biological components of parenthood. However, our legal and conceptual frameworks do not seem to have kept pace. Hence the assumption that the genetic and gestational mothers must be the same, and that genetic fatherhood is inextricably tied up with legal and financial responsibilities. But since women have the right to alienate legal parental ties to their offspring, perhaps we should extend the same courtesy to men in some circumstances.

It would seem unjust both to overrule Mr Johnston's wishes with regard to the implantation of the embryos *and *to demand of him financial and legal responsibility for the offspring. However, if a man in these circumstances were able to revoke his legal responsibilities, in the way that a sperm donor can, his grounds for complaint over enforced genetic fatherhood would be far less obvious.

### Comparisons with Davis v. Davis

It is interesting here to compare the Evans case with that of Davis v. Davis in the US. The couple in question parted and a dispute arose over embryos in storage[9]. As in the Evans case, the embryos were eventually destroyed. The negative consequences of forced fatherhood were described, especially their financial and psychological impact. In such cases, it was suggested, '... the party wishing to avoid procreation should prevail'[10]. Similarly, Mr Johnston's lawyer emphasised the psychological and financial impact of unwanted genetic parenthood. If Ms Evans had gone ahead against his wishes, Mr Johnston 'on a biological and psychological basis [...] would be the father of the child for whom he would have ongoing moral, legal and financial responsibilities'[11].

The men – and the courts – in both these cases seemed to assume that the financial burdens and the psychological pressure of forced parenthood were inextricably linked with the birth of a genetically-related child. However, this is not necessarily the case since sperm donors do not have financial obligations toward their offspring. If the possibility of allowing Mr Johnston to revert his legal status to that of a sperm donor had been addressed, a more thorough analysis of any further objections would have been possible. Specifically, the issue of psychological suffering could be considered without being confused with the question of financial responsibility.

Mr Johnston felt that simply by virtue of sharing some of his genes, a child born to Ms Evans would impose a psychological burden on him. Even if he could be legally exempt from any financial or legal obligations, he believed that the existence of a biological link conferred an inalienable parental bond. Rather than submit himself to such a bond, he preferred that the embryos which contained his genes should be destroyed.

The gestational or financial components of parenthood may cause obvious and objective harm when forced on unwilling people. But psychological pain caused solely by the existence of genetically-related children is far more subjective. The fact that some people choose to donate sperm or eggs demonstrates that not everyone feels the same way about this. Any moral or psychological harm involved solely in becoming a genetic parent is contingent on the personal beliefs of the adult involved.

Clearly, Mr Johnston's beliefs made him strongly averse to the prospect. But it is questionable how much credence such a view should be given. Ms Evans had her own equally subjective view about the value to her of genetic parenthood. It may well be that both parties placed too much weight on this account of parenthood and its implications.

### Divisions of genetic parenthood

Genetic parenthood is widely perceived as a simple and unassailable biological fact. This was at the root both of Mr Johnston's and Ms Evans' perceptions of the importance of controlling the disposal of embryos which contained some of their genetic material. However, just as in vitro technology has allowed for the separation of gestational and genetic motherhood, the integrity of genetic parenthood itself may be challenged by further developments in reproductive technology[12].

Some women are known to have genetic abnormalities in their mitochondrial DNA. These women may transmit these abnormalities to their offspring, causing disease, disability and sometimes death. It has been suggested that this could be overcome by removing the nucleus from the affected mother's fertilised egg and inserting it into an empty egg cell donated by a woman whose mitochondrial DNA is normal. The resulting child would be free of mitochondrial disease. ... but should either, or both of these women be properly regarded as the genetic mother[13]?

Similar questions are raised by the prospect of in vitro derived gametes. It has been suggested that, for example, an enucleated egg could be injected with the nucleus of an adult's skin cell[14,15]. The inner cell mass would then be cultivated and embryonic stem cells derived. These could then be differentiated into sperm or egg cells as required. A child born from sperm and eggs derived in this way could also inherit DNA from three individuals (mitochondrial DNA from the egg donors, and chromosomes from two nucleus donors).

These possibilities reveal the complexity behind the apparently simple truth that reproduction requires exactly two genetic parents. Biological boundaries are more fluid than we tend to imagine. Reproductive technology should have taught us this. With the possibility of IVF and surrogacy, concepts that were thought to be simple biological facts turned out to be far more complex. This is likely to continue with further research, extending to the understanding of genetic parenthood. This fluidity needs to be mirrored, as far as possible in the law and in people's perceptions.

Changing perceptions of genetic parenthood is unlikely to be an easy or quick process. However, there are a number of lessons to be learnt from the case which may point the way to improvements in avoiding such disputes, and to dealing better with them when they do arise.

### Improving the circumstances around obtaining consent

It has been pointed out that the quality of Ms Evans' and Mr Johnston's initial consent was deeply flawed[16]. The time allowed to the couple to make their decision was limited, and they were understandably distressed at the diagnosis of Ms Evans' condition. Mr Johnston may have felt unwilling to add to Ms Evans' burdens by expressing doubts as to the permanence of their relationship. Uncertain of his own commitment, he may have felt that the best option was to buy time with the creation of the embryos: *he knew he could always withdraw his consent later*.

Clinics should be aware of the difficulties faced by couples in this situation. In particular, offering separate consultations could be helpful. Where feasible, decisions should not be taken at a time of additional stress. It is also important that all the options available are considered. If Ms Evans had frozen her eggs instead of creating embryos, the subsequent problems could have been avoided. Some clinics may hesitate to recommend egg freezing on account of its low success rate. However, it should be borne in mind that some women might prefer the risk of unsuccessful treatment to the risk of being unable to use embryos created with a partner's sperm. Another possibility would be to offer women in Ms Evans' circumstances the chance to fertilise some of her eggs with donated sperm. The essential thing is to try to ensure that both parties have considered all the options that are open to them, together with any associated risks and benefits.

Timing is also important. Ms Evans was advised to wait two years before having the embryos implanted. During this period, her relationship broke up. Had she implanted the embryos immediately, Mr Johnston would not have been able to thwart her parental endeavours. The advice to wait was founded on clinical concerns. However, again clinics need to be aware that a patient's social interests may not necessarily tally with her best medical interests. The implications of delaying implantation should be addressed, and should be identified as a risk in the event of a break-up.

In the UK, the Human Fertilisation and Embryology Authority (HFEA) provides standard consent forms for the creation and storage of embryos. Separate forms are provided for the man and woman. The forms require details on how long embryos can be stored, and what should be done with them if the individual dies or loses capacity. However, no specific reference is made to the disposition of embryos in the event of a relationship breakup.

Where couples are creating embryos together it would be advisable to recognise that they are seeking treatment *as a couple*. The issues facing couples are very different from those facing single people, and their concerns cannot be encompassed by consent forms that do not recognise the potential for conflict. This need not mean that couples have to reach unanimous decisions about the disposition of their embryos. But they should specify what happens to embryos in the event of a breakup as well as in the event of death or incapacity, as in the current forms.

### Resolving disputes

However careful clinics and prospective parents are, they cannot obviate every dispute. So when, despite all precautions, conflicts arise, how should they be dealt with?

It has been argued that women should be regarded as having a greater investment in the embryos. This might seem justified in terms of the greater physical burdens that fertility treatment imposes on them[17]. It could also reflect the point made above: men's willingness to create embryos may represent a more conditional step toward parenthood than it does in women. Moreover, while sperm can be frozen and used with little reduction in efficacy, egg-freezing is much less likely to result in a succcesful birth. On almost every level, women in Ms Evans' position are at a disadvantage relative to men.

Yet prioritising the female partner's stake in disputed embryos would not necessarily improve outcomes for women in general. Men might be more reluctant to create embryos in the first place if their consent were taken to be less than binding. And if women are understood to have a greater interest in the disposal of embryos, this could be seen as a reactionary move to emphasise women's role as mothers over other interests they may have. (Nevertheless, an Israeli court has made a judgement based on this perception of a woman's greater interest in being a mother[18].)

If neither sex is to be systematically favoured, there seems little option but to revert to the consent provisions. In theory, this should prevent any unjust elevation of one person's interests over the other. However, there is a strange anomaly here. The consent of both parties is required for the 'use' or ongoing storage of the embryos, yet they can be destroyed with the consent of only one party. This asymmetry has been severely criticised: " [s]urely if rights were equal the law would be prevented from doing anything with the embryos unless the parties were in agreement"[19].

Seemingly, the parties' interests are *not *being given equal consideration. Those who wish to become parents are systematically disadvantaged since they require two consents, whereas those who want to avoid parenthood can do so with ease simply by withdrawing their own consent.

There may be pragmatic reasons for the discrepancy. If destruction were not the default option, embryos' legal status would be indeterminate. Ongoing storage would be unlawful and they could neither be used for fertility treatment, nor destroyed, nor used in research. For this reason, it is commonly assumed in such cases that destruction of the embryos is an unfortunate necessity.

Since embryos can thus be destroyed on a unilateral basis, this should surely be highlighted in the HFEA's standard consent forms. However, it is not[20]. Nor is it stipulated in the HFE Act itself. The fact that both parties' consent is required for ongoing storage and for any use of embryos, is simply taken to *imply *that destruction is the default option in the event of a dispute. But patients signing such a form cannot necessarily be expected to make this inference unaided. In the broader context of medical ethics, it is generally accepted that if a patient consents to X, and X entails Y, the patient cannot thereby be assumed to have consented to Y unless specifically informed of this entailment[21].

The vagueness in the law and in the consent forms is a serious problem. Patients who are made aware that ongoing consent of both parties is required for the use of their embryos, may assume incorrectly that the same would be required for their destruction.

Even if it is pragmatically justified *and *thoroughly understood by both parties, the destruction of embryos as a default measure may have very different consequences for the individuals involved. Mr Johnston retained the option of having genetically-related children at another time in his life. For Ms Evans the embryos' destruction precluded any chance of genetic parenthood. The reasoning behind the requirement of consent from both parties is that their interests have equal weight. But can this equality be achieved when the destruction of embryos carries such different implications for the parties involved, and can be carried out at the behest of only one of their progenitors?

The ruling in Davis v. Davis left scope for a different outcome if the disputed embryos represent one party's only chance of having genetically-related offspring. Although the wishes of the person who does not want to become a parent should generally be paramount, this was based on the assumption that 'the other party has a reasonable possibility of achieving parenthood by means other than use of the embryos in question'[22].

If any circumstances might justify this kind of exception, surely those of Ms Evans did. But in a context where the genetic, financial, legal and moral conceptions of parenthood are so confused, it is perhaps unsurprising that the compassionate response was not forthcoming. Legally, the outcome was to be expected: Ms Evans could not demonstrate that her rights had been violated.

The case of Ms Evans shows that even where there is the technological ability to remedy infertility, social and legal concerns may nevertheless override a woman's desire to become a mother. This is an important consideration to bear in mind: technology alone cannot solve problems which are bound up with social and legal proscriptions. It is not always possible to foresee the social and legal complexities that may arise from apparently benign techniques.

## Summary

This case raises questions about the nature of genetic parenthood, and what this entails in law. It also raises questions about popular perceptions of parenthood, and the degree to which these are necessarily associated with genetic parenthood as such. In legal terms, it may have made sense in the pre-IVF era to focus on genetic ties in order to establish parental responsibility. However, in a world where the biological components of parenthood have become fragmented[23] and where women and men can earn broadly comparable salaries, it no longer makes sense to base paternal responsibility solely on genetic criteria[24].

Legal and social flexibility is required in order to accommodate the challenges of developments in reproductive technology. This is unlikely to be resolved in the near future. Therefore it is vital that consent procedures address the possibility of relationship breakup, and inform couples of the fact that embryos will be destroyed in the event of a dispute.

## Competing interests

The author(s) declare that they have no competing interests.
